# Effects of tendon transfer to restore index finger abduction for severe cubital tunnel syndrome

**DOI:** 10.1080/03009730802702602

**Published:** 2009-04-24

**Authors:** Shingo Nobuta, Katsumi Sato, Kenji Kanazawa, Masahito Hatori, Eiji Itoi

**Affiliations:** ^1^Department of Orthopaedic Surgery, Tohoku Rosai HospitalSendai MiyagiJapan; ^2^Department of Orthopaedic Surgery, Tohoku University School of MedicineSendai MiyagiJapan

**Keywords:** Cubital tunnel syndrome, extensor pollicis brevis, index finger abduction, tendon transfer

## Abstract

**Background:**

Severe cases of cubital tunnel syndrome do not always result in functional recovery after surgical decompression of the ulnar nerve. A combined operation of tendon transfer to restore index finger abduction and decompression of the ulnar nerve was performed for patients with severe cubital tunnel syndrome who required powerful pinch strength and whose preoperative compound muscle action potential of the abductor digiti minimi muscle was not recordable or almost non-recordable. The purpose of this study was to assess the efficacy of tendon transfer to restore index finger abduction for severe cubital tunnel syndrome.

**Methods:**

Sixteen hands in 15 patients with severe cubital tunnel syndrome were operated on with extensor pollicis brevis tendon transfer to the first dorsal interosseous muscle to restore index finger abduction and ulnar nerve decompression. They were reviewed after a mean follow-up of 16 months. All 16 hands had preoperatively severe lesions with paralysis of ulnar intrinsics, marked anaesthesia, or hypaesthesia.

**Results:**

Postoperative results were excellent in 2 hands, good in 10, fair in 4, and no cases with poor results according to Akahori's criteria. Four hands with fair results had a residual Froment sign or annoying hypaesthesia in the ring and little fingers. All patients were relieved of preoperative discomfort and showed recovery of motor and sensory function. The mean pre- and postoperative pinch strength was 3.3 kg and 5.6 kg, respectively, which showed a significant difference (*P*<0.01). Mean time of showing a negative Froment sign after surgery was 5 months in 13 cases.

**Conclusions:**

The extensor pollicis brevis tendon transfer is simple and useful to restore index finger abduction and pinch strength for severe cubital tunnel syndrome.

## Introduction

Ulnar neuropathy at the elbow is the second most common peripheral nerve compression neuropathy (1). Cubital tunnel syndrome is secondary to posttraumatic skeletal deformities or to disorders of the elbow (2). Previous studies have reported good clinical results for cubital tunnel syndrome after surgical decompression of the ulnar nerve (3–7), but severe cases with intrinsic muscle atrophy and weakness do not always result in functional recovery (8–14). The interval between operation and recovery in cubital tunnel syndrome was reported to be more than one year (15), two years (16), or more than two years (17,18). We reported a trend toward poor functional results in patients with non-recordable compound muscle action potential (CMAP) of the abductor digiti minimi (ADM) muscle before surgery, and that the mean interval between surgical decompression and functional recovery of intrinsic muscles in the recovered cases was 19 months (19). From 2002, we performed a combined operation of the extensor pollicis brevis tendon transfer to restore index finger abduction reported by Bruner (20) and decompression of the ulnar nerve for patients who required powerful pinch strength from the early postoperative phase and whose preoperative ADM-CMAP was not recordable or almost non-recordable. The purpose of this study was to assess the efficacy of Bruner's tendon transfer for severe cubital tunnel syndrome.

## Patients and methods

Between October 2002 and January 2008 a total of 21 hands in 20 patients with severe cubital tunnel syndrome underwent operations with Bruner's tendon transfer and ulnar nerve decompression. Sixteen hands in 15 patients were followed up for 6–26 months (mean: 16 months) and enrolled into this study. The details are shown in Table I. Twelve patients were men and three were women; a male patient had bilateral lesions (cases 11 and 13). The age at operation was 45–77 years (mean: 66 years). The duration of symptoms had varied from 3 to 25 months, the mean being 13 months. All patients were associated with osteoarthritis of the elbow joint. The dominant extremity was involved in 12 patients and the non-dominant extremity in 2 (cases 4 and 12); one patient had bilateral lesions. All cases had a positive Tinel sign at the cubital tunnel, a positive Froment sign, and marked atrophy of intrinsic muscles with clawing of the ring and little fingers. There were hypaesthesia to light touch in the above-mentioned fingers in 12 patients, and anaesthesia in 4. Preoperatively, all cases were classified into grade 3 (severe lesions with paralysis of one or more of the ulnar intrinsic muscles) according to McGowan's classification (8). Twelve hands were stage 4 (Froment sign, atrophy of intrinsic muscles, and clawing of fingers), and four were stage 5 (clawing of fingers and absent sensation) according to Akahori's classification (5). Recording and analysis of ADM-CMAP as a nerve conduction measurement were performed in all cases before surgery. If ADM-CMAP was not recordable, CMAP of the flexor carpi ulnaris (FCU) muscle was recorded and analysed for definite diagnosis of cubital tunnel syndrome. Even in severe cases, FCU-CMAP is recordable because the FCU branch is separate and rarely involved in cubital tunnel syndrome (11).

**Table I.** Details of 16 hands in 15 patients.

						Measurements before/after surgery			
Case	Age/Sex (yr)	Side	Duration of Symptoms (mth)	Stage of severity	Follow-up (mth)	Grip (kg)	Pinch (kg)	Time of negative Froment sign after surgery (mth)	Result
1	61/M	R	5	4	23	21/23	2.8:3.5	7	Fair
2	45/M	R	16	5	26	35/50	7.0/9.5	2	Excellent
3	66/M	R	24	5	25	15/24	3.0/6.5	2	Excellent
4	59/M	L	5	4	18	13/28	2.4/6.8	3	Good
5	55/M	R	15	4	16	28/31	3.4/5.5	3	Good
6	77/F	R	10	4	12	5/10	3.9/4.4	1	Good
7	70/M	R	16	4	25	14/15	0.1/4.5	3	Good
8	59/M	R	10	4	17	24/30	0.5/7.2	6	Good
9	67/M	R	19	4	15	20/26	3.4/6.9	(+)	Fair
10	68/M	R	5	4	18	17/25	4.8/4.9	(+)	Fair
11	72/M	L	12	5	14	6/15	5.5/6.0	9	Good
12	74/M	L	25	4	12	26/28	2.1/3.5	3	Good
13	72/M	R	24	5	12	13/21	6.5/7.6	12	Good
14	73/F	R	13	4	12	10/18	1.9/3.3	(+)	Fair
15	63/M	R	3	4	10	27/35	4.0/6.4	6	Good
16	75/F	R	6	4	6	7/9	1.4/2.0	4	Good

Stage of severity according to Akahori's classification. M = male; F = female; R = right; L = left; (+) = Froment sign was positive at the time of follow-up.

**Figure 1.**
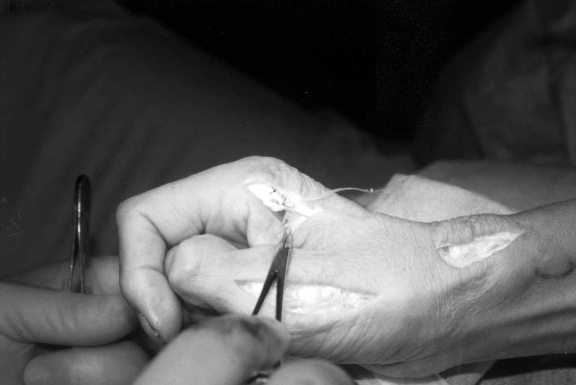
The extensor pollicis brevis tendon is freed at the metacarpo-phalangeal joint of the thumb, and withdrawn to the proximal end of the anatomical snuff-box. The re-routed extensor pollicis brevis tendon is sutured to the tendinous portion of the first dorsal interosseous muscle near its insertion.

Fourteen cases underwent decompression of the nerve and medial epicondyle excision (21), and two had simple decompression of the nerve (22). All cases underwent Bruner's tendon transfer. The extensor pollicis brevis tendon was freed at the dorsal surface of the metacarpo-phalangeal joint of the thumb and withdrawn to the proximal end of the anatomical snuff-box. It was then re-routed subcutaneously under the extensor pollicis longus tendon, toward the insertion of the first dorsal interosseous (FDI) muscle. The re-routed extensor pollicis brevis tendon was sutured to the tendinous portion of the FDI muscle near its insertion (Figure 1). After surgery, the hand and forearm were held in a plaster splint with the index finger in slight abduction and the wrist in slight extension. Physical therapy was started after three weeks with caution against strong ulnar flexion of the index finger until six weeks.

The results were evaluated into four categories according to Akahori's criteria (5). Excellent was complete or almost complete recovery. Good meant that the patient had recovery of motor function with a negative Froment sign and clawing of fingers with some residual complaints. Fair was slight improvement with no functional recovery. Poor was no benefit from the operation. Grip and pinch strength were measured before and after surgery. Statistical analysis was performed with Student's *t* test, and a *P*-value of less than 0.05 was considered significant.

## Results

**Figure 2.**
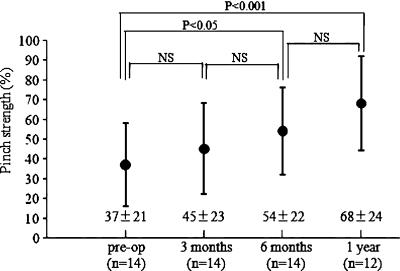
Changes in pinch strength, gaining a significant increase at 6 months after surgery compared with preoperative pinch strength.

The results were excellent in two cases, good in ten cases, fair in four cases, and no cases showed a poor result (Table I). Preoperative ADM-CMAP was not recordable in ten cases and recordable in six (cases 3, 6, 9, 12, 13, and 14). Amplitude of ADM-CMAP was 1% compared with the normal side in cases 3 and 9, and 6% in cases 6, 12, and 14. Cases 11 and 13 were from one patient with bilateral lesions, and the amplitude was 0.1 mV in case 13. In four cases of fair results, case 1 had residual annoying hypaesthesia and numbness in the ring and little fingers. Cases 9, 10, and 14 had a residual positive Froment sign at the time of follow-up. However, all cases were satisfied with the results, relieved of preoperative discomfort, and showed improvement in motor and sensory function, especially in grip and pinch strength. The mean preoperative grip and standard deviation (SD) was 17.6±8.8 kg, and the mean postoperative grip and SD was 24.3±10.2 kg, which revealed no significant difference. The mean preoperative pinch strength was 3.3 kg (SD 2.0 kg), and the mean postoperative pinch strength was 5.6 kg (SD 1.9 kg), which showed a significant difference (*P <* 0.01). In the 14 unilateral cases, pinch strength significantly increased from 37% (SD 21%) of that of the normal side preoperatively to 54% (SD 22%) at 6 months after surgery (*P <* 0.05), and 68% (SD 24%) at one year after surgery (*P <* 0.001) (Figure 2). The time of showing a negative Froment sign (13 cases) was 1–12 months after surgery, the mean being 5 months. There were no cases with weakness of extension on the metacarpo-phalangeal joint of the thumb, and no cases with instability of the thumb after freeing the extensor pollicis brevis tendon. No complications were observed.

## Discussion

The indication for tendon transfer to restore index finger abduction for severe cubital tunnel syndrome is still controversial. Shiraishi et al. (16) described that 6 of 20 cases were fair or poor in which ADM-CMAP was not recordable, and the duration of preoperative symptoms was 10 years or more. They showed that five patients who had undergone the tendon transfer to restore index finger abduction were satisfied with functional recovery. Yokomura et al. (17) reported that 18 of 32 cases had fair or poor results and concluded that reconstructive surgery was indicated if ADM-CMAP was not recordable or functional recovery was not seen at 2 years after surgery. Futami et al. (23) reported that the tendon transfer was indicated for patients who required secure recovery of pinch function and whose marked atrophy of the FDI muscle persisted for more than 2 years. Nakagawa et al. (12) showed indication of restoration of index finger abduction for patients in which the preoperative FDI muscle power was zero. Froimson et al. (4) reported that two of six cases with severe lesions required tendon transfers to improve function. Matsuzaki et al. (18) reported that restoring abduction of the index finger by tendon transfer was not always necessary, but it was indicated for older patients who required strong pinch force soon after surgery.

The FDI muscle function is necessary for index finger abduction, and the index is the finger against which the thumb is brought most frequently in pinch movement (24). The FDI muscle is often more denervated than the ADM muscle (6,25). We reported a postoperative poor function in patients with non-recordable ADM-CMAP and showed that the mean interval between surgical decompression and functional recovery of intrinsic muscles was 19 months (19). Therefore, we believe that a combined operation of tendon transfer to restore index finger abduction and decompression of the ulnar nerve is indicated for patients who require powerful pinch strength from the early postoperative phase and whose preoperative ADM-CMAP is not recordable or almost non-recordable.

Bruner (20) described that the extensor pollicis brevis tendon had a favourable angle of pull, was long enough to be used without lengthening by graft, and was strong enough to restore useful abduction of the index finger. Takeda et al. (26) showed the results of Bruner's procedure for six cases and described that the mean postoperative pinch strength was 2.76 kg and the mean time of functional recovery was 3.1 months after surgery. In our 16 cases, all had improvement in grip and pinch strength, even in 3 cases with a residual positive Froment sign. The mean postoperative pinch strength was 5.6 kg and significantly increased at 6 months after surgery. The mean time of showing a negative Froment sign after surgery was 5 months, which was almost similar to a previous report (26). A Froment sign is shown when the flexor pollicis longus plays a part of the paralysed adductor pollicis in the pinching function, and a negative Froment sign means that abduction of the index finger is useful in the pinch movement.

With regard to other procedures of tendon transfer to restore index finger abduction, Neviaser et al. (27) reported abductor pollicis longus transfer for replacement of the first dorsal interosseous. Saito et al. (28) showed that postoperative pinch strength was 7.7 kg after surgery with Neviaser's procedure. Futami et al. (23) described the results of Neviaser's operation in 14 cases and showed that pinch strength recovered in all cases at about 3 months after operation. Nemoto et al. (29) reported Neviaser's procedure for 18 cases and stated that the mean postoperative pinch strength was 75% of that on the normal side at one year after surgery. In Neviaser's procedure, the abductor pollicis longus tendon is reported to be stronger than the extensor pollicis brevis tendon (26), and postoperative pinch strength after Neviaser's procedure (28,29) was stronger than that of our series. However, the abductor pollicis longus tendon is not long enough to be used and needs lengthening by tendon graft to suture to the tendinous portion of the FDI muscle (24). Nemoto et al. (29) reported that the extensor pollicis brevis was better spared because the muscle played an important role in stabilizing the interphalangeal joint of the thumb. However, in our series, there were no cases with instability of the interphalangeal joint or weakness of extension on the metacarpo-phalangeal joint of the thumb after surgery. The extensor pollicis brevis tendon is long enough to be used without lengthening by graft (20), but this tendon may be absent (2 of 52 hands) and may be too thin for the expected new function (30). Therefore, surgeons should confirm that the tension of the extensor pollicis brevis tendon is sufficient for reconstruction before surgery. For most cases with severe cubital tunnel syndrome, Bruner's tendon transfer is simple and useful to restore index finger abduction and powerful pinch strength soon after surgery.

## Conclusions

Bruner's tendon transfer is simple and useful to restore index finger abduction for severe cubital tunnel syndrome.
